# Step-Specific Adaptation and Trade-Off over the Course of an Infection by GASP Mutation Small Colony Variants

**DOI:** 10.1128/mBio.01399-20

**Published:** 2021-01-12

**Authors:** Christian Faucher, Vincent Mazana, Marion Kardacz, Nathalie Parthuisot, Jean-Baptiste Ferdy, David Duneau

**Affiliations:** a CNRS, UMR5174 EDB (Laboratoire Évolution & Diversité Biologique), Université Toulouse 3 Paul Sabatier, Toulouse, France; b Instituto Gulbenkian de Ciência, Oeiras, Portugal; Northern Arizona University

**Keywords:** host-parasite interactions, steps of infection, within-host evolution, bacteria, *Drosophila*

## Abstract

During an infection, parasites face a succession of challenges, each decisive for disease outcome. The diversity of challenges requires a series of parasite adaptations to successfully multiply and transmit from host to host. Thus, the pathogen genotypes that succeed during one step might be counterselected in later stages of the infection. Using the bacterium Xenorhabdus nematophila and adult Drosophila melanogaster flies as hosts, we showed that such step-specific adaptations, here linked to GASP (i.e., growth advantage in stationary phase) mutations in the *X. nematophila* master gene regulator *lrp*, exist and can trade off with each other. We found that nonsense *lrp* mutations had lowered the ability to resist the host immune response, while all classes of mutations in *lrp* were associated with a decrease in the ability to proliferate during early infection. We demonstrate that reduced proliferation of *X. nematophila* best explains diminished virulence in this infection model. Finally, decreased proliferation during the first step of infection is accompanied by improved proliferation during late infection, suggesting a trade-off between the adaptations to each step. Step-specific adaptations could play a crucial role in the chronic phase of infections in any disease organisms that show similar small colony variants (SCVs) to *X. nematophila*.

## Abstract (precis)

Within-host evolution has been described in many bacterial diseases, and the genetic basis behind the adaptations has stimulated a lot of interest. Yet, the studied adaptations are generally focused on antibiotic resistance and rarely on the adaptation to the environment given by the host, and the potential trade-offs hindering adaptations to each step of the infection are rarely considered.

## INTRODUCTION

Successful colonization of a host is essential to the life cycle of pathogens. Over the course of an infection, pathogens face a series of barriers to establishing an infection, each one decisive for the outcome. Depending on the system and on our level of resolution, we can distinguish a variable number of such steps. In the simplest case, parasites need to first encounter and attach to their host, then to overcome the different lines of immune defense to proliferate and establish within the body, and finally, to transmit to another host (1–3). Each transition between two steps imposes a new challenge that pathogens might overcome with step-specific adaptations. If traits involved in different steps are uncorrelated, independent adaptative mutations may occur and increase in frequency until the pathogen succeeds at each and every step of the infection. But those step-specific adaptations may also trade off with each other: the pathogenic strains that are successful and dominant at the initiation of the disease might differ from those that are successful and dominant at a given advanced step of an infection. Within-host evolution has been described in many human bacterial diseases, such as those associated with Helicobacter pylori (4), Staphyloccocus aureus (5), or the causative agent of melioidosis, Burkholderia pseudomallei (6), and the genetic basis behind the adaptations has stimulated a lot of interest. However, the studied adaptations are generally focused on antibiotic resistance and rarely on the adaptation to the environment given by the host, and the potential trade-offs hindering adaptations to each step of the infection, especially within the host, are rarely considered (7).

Such trade-offs limit our understanding of how pathogens evolve until we precisely describe the way they interact with their hosts at each step of the disease process. The bacterium Salmonella enterica is a good example of a model in which trade-offs have been described (8). The *in vivo* adaptation to a specific host trades off with the transmission to another host (9), and the adaptation to remain in the blood trades off with the ability to infect the gastrointestinal environment by the loss of key gene functions (10). In Salmonella enterica, phase variation (i.e., a mechanism for high-frequency back-and-forth switching between phenotypes) of the virulence factor impeding fast proliferation over the course of the infection has been selected to avoid this trade-off (11), but this mechanism is not present in all bacterial diseases, and it is not clear whether bacterial adaptations for each step are impeded by the trade-off.

The bacterial entomopathogen Xenorhabdus nematophila is a tractable model that can help in understanding the consequences of step-specific adaptations on intrahost evolution. In the wild, *X. nematophila* forms a symbiotic association with its vector, the nematode Steinernema carpocapsae, which lives in soil and reproduces in insect hosts. Once in an insect gut, the *S. carpocapsae* vectors inject a few cells of their bacterial symbiont into the insect hemolymph. The bacterial population proliferates despite the host immune response, produces toxins to kill it rapidly, and degrades host tissues, which in turn supports the nematode’s development and reproduction inside the host body cavity. After the insect's death, the bacteria cannot disperse before the population of nematode vectors grows. At this step of the infection, the bacteria and nematode must share the dead insect as a stock of resources that are no longer replenished; nematodes then often eat the bacteria to ensure their survival (12). Once dispersing nematode offspring are produced, a small number of bacteria associate with them, thanks to a very specific set of genes, and disperse with the vector (13–16). Thus, we can recognize at least three discrete steps in the infection (17): (i) one where the bacteria are in a dedicated vesicle of the nematode, waiting for the next infection; (ii) one where the bacteria need to survive the insect immune response and destroy host tissue for the establishment of their vector; and (iii) a final step where the bacteria need to persist in the decaying insect host to eventually feed and colonize the vector.

For decades, the entomopathogen bacteria Xenorhabdus nematophila has been described as occurring under two phenotypes resulting from phase variation (18). Bacteria from the phenotype often referred as to as phenotype 1 are mobile/flagellated and produce antibiotics, hemolysins, immune suppressors, and toxins, while bacteria from phenotype 2 generally lack these characteristics (19, 20) and are about 10 times smaller (7). However, it has recently been shown that those two discrete phenotypes are in fact not due to phenotypic switching but to selection during the infection of mutations in the leucin-responsive regulatory protein gene (*lrp*) (7). Even though a variety of *lrp* mutations are known to be selected over the infection, the differences in advantage they could confer is unknown. *lrp* is a strongly conserved global transcriptional regulator widely distributed among prokaryotes and archaea (21–23). In Escherichia coli, it is involved in amino acid metabolism, monitors the general nutritional state by sensing the concentrations of leucine and alanine in the cell, and regulates genes involved in entering the stationary phase of growth (24, 25). In fact, *lrp* controls the gene expression of about a third of the genome (26) and acts as a virulence regulator in numerous infectious diseases, including those caused by Salmonella enterica serovar Typhimurium (27), Vibrio cholerae (28, 29), mycobacteria (30, 31), Clostridium difficile (32), and Xenorhabdus nematophila (33, 34). By comparing *in vitro* phenotypes linked to the presence or absence of the mutation in *lrp* with the cycle of the bacteria, one can make a clear hypothesis on how the mutants are selected over the course of the infection. The characteristics of the strains without mutation in *lrp*, described above, suggest that these bacteria are selected to invade a living host carrying other bacterial competitors that must be eliminated to prepare the environment for the nematode. Consistent with this hypothesis, in nature, nematodes generally carry a clonal bacterial population with phenotype 1, which we name below as the “wild-type” strain (18). On the other hand, the typical characteristics of bacteria living in an environment limited in resources—stationary phase (35)—correspond to the characteristics of the bacteria in phenotype 2, below named “mutant.” This suggests that mutations in *lrp* could give an advantage to wait in a decaying host, where the quality and/or quantity of resources or other conditions, such as pH, change. In fact, *lrp* mutants outcompete the wild type in aged *in vitro* cultures of *X. nematophila* (7), as does Escherichia coli (36), a phenotype described as growth advantage in stationary phase (GASP) (37). Furthermore, *lrp* mutants can outcompete the wild type *in vivo*, when they colonize mouse gut (38). Thus, a clonal bacterial population selected to initiate the infection could be first favored until the environment changes and mutants able to grow under stressful conditions become dominant. These mutants can potentially associate with the nematodes, even though badly, but they have never been found in wild-caught nematodes. However, they may serve as food or process available food to provide nutrients for their vector until the rare wild-type genotype or a genotype with a compensatory mutation, allowing phenotype reversion, can reassociate with the nematodes, disperse from the host cadaver inside the new vector, and initiate a new infection. It is not clear whether acquiring this adaptation to persist in the host would hamper the adaptation to initiate infection.

In this study, we investigated the bacterial adaptations resulting from mutations known to be positively selected within the host over the course of an infection and the trade-off between step-specific adaptations. More specifically, we characterized the consequences of nonsense and missense mutations in the major regulator gene *lrp* on infection of Drosophila melanogaster at different steps of the infection. Our results suggest that virulence decreased as mutations in *lrp* become more disruptive to gene function, correlating with the ability to grow at the beginning of the infection. Despite the fact that bacteria carrying nonsense mutations in *lrp* proliferated better in immunodeficient (IMD) compared to healthy hosts, the ability to cope with immune system activation did not correlate with virulence. Furthermore, mutants killed hosts at similar bacterial loads to the wild type, suggesting that they were as pathogenic as wild-type strains and that the ability to kill was solely explained by the speed of proliferation within the host. Next, we demonstrated that mutants, regardless of the type of mutation, were well adapted to the waiting step of the infection as they proliferated better in dead hosts than does the wild type. Our results suggest that wild-type strains perform better than mutants during the first step of infection, but less well during the second step. We demonstrate that as the infection progressed, the ability to grow well in the dead host was acquired, while the ability to grow well in the healthy host was lost, suggesting a trade-off between the adaptations to each step.

## RESULTS

### The bacterium-host model.

**FIG 1:**
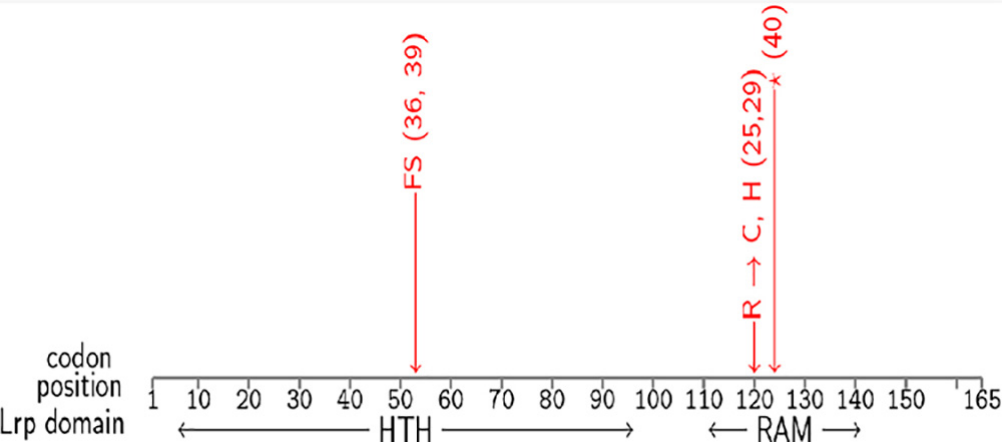
Description of *lrp* mutations. The *lrp* gene is made of two domains, HTH and RAM. The study used four *lrp* mutants, each with a single point mutation that strains 23, 42, 44, and 51 do not carry. Strains 36 and 39 carry different nonsense mutations in codon position 53, leading to a frameshift (FS). Strains 25 and 29 carry each a missense mutation in codon position 120, leading to different amino acids. Strain 40 carries a nonsense mutation (duplication) in codon position 124, leading to a Stop codon (*).

Xenorhabdus nematophila is a generalist and highly virulent entomopathogen bacterium. It is generally described as having two distinct phenotypes, distinguished in the lab by their capacity to adsorb the dye bromothymol blue. This phenotype is associated with mutations in the *lrp* gene occurring *in vitro* and *in vivo*, which the wild-type strains do not carry. The strains used in this study were chosen from a collection of 34 strains: some are wild type for the *lrp* gene (shown in blue in the following figures), while the others carry various classes of mutation in different domains of *lrp* (shown in red in the following figures). The *lrp* mutations are illustrated in Fig. 1. D. melanogaster is not a natural host of our generalist parasite, but the genetic tools this model offers allowed us to test predictions on the role of arthropod host immunity in the success of wild-type and mutant bacteria (39–41).

### *lrp* mutations impede virulence.

**FIG 2:**
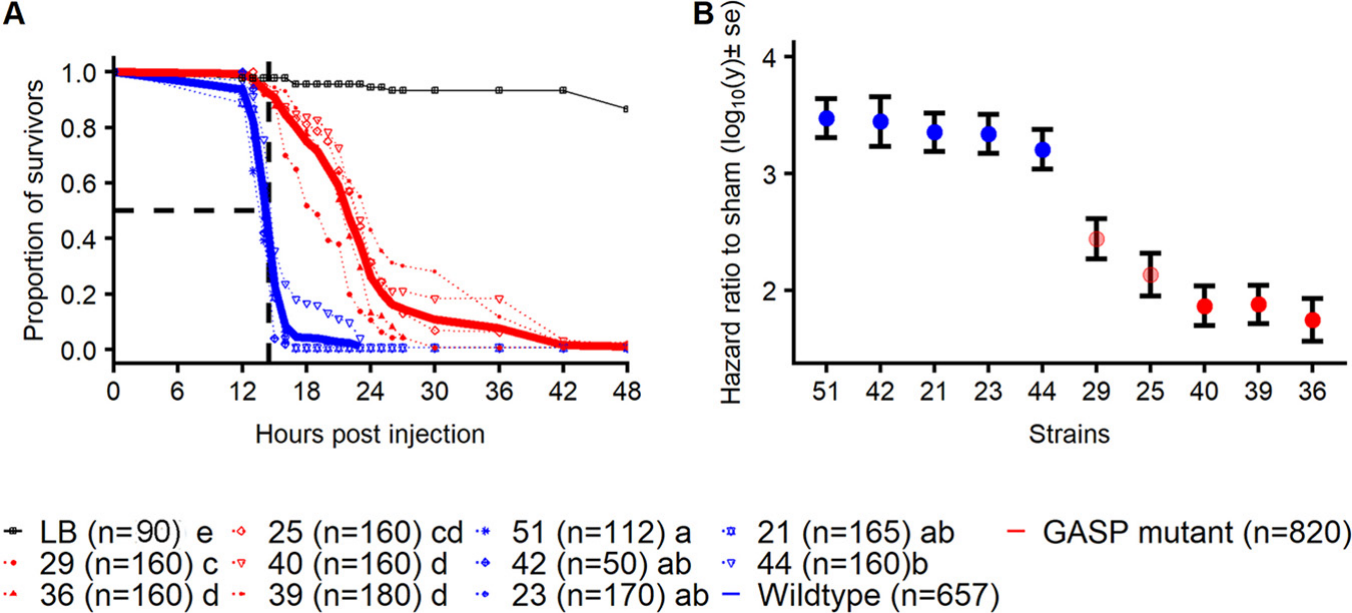
Survival of adult Drosophila melanogaster flies upon infection with wild-type or *lrp* mutant Xenorhabdus nematophila. (A) Survival over time of hosts when injected with 1,000 bacteria. Wild-type bacteria, represented individually by blue dotted lines, always killed their hosts faster than *lrp* mutants, represented individually by red dotted lines. Solid lines represent the pooled wild-type (blue) and mutant (red) strains. Black dashed lines show the median lethal time in hours (LT_50_) of wild-type strains. The numbers of host individuals are mentioned in parentheses. (B) Virulence of each bacterial strain. Each dot represents the death hazard ratio relative to sham infection calculated from the Cox model used to analyze the survival in panel A. Blue represents infection with wild-type bacterial strains, and red represents infection with mutant strains. Details of strain numbers for reference are given in Fig. 1.

We investigated the difference in virulence between *X. nematophila* strains. Using Canton S wild type strains as the host genotype, we found that all strains killed their host in less than 2 days postinjection, but flies infected with wild-type bacteria died more rapidly than those infected with mutants (coxme, χ^2^ = 21.4, *P* = 3.7e−06 [Fig. 2A]). Where wild-type bacteria killed on average 50% of their hosts in approximately 14 h, mutants killed almost none of their hosts (∼7%). We computed a hazard ratio relative to sham infection (Fig. 2B): the risk of death was increased on average by a factor of 10^3.3^ when hosts were infected by wild-type strains, but only by a factor of 10^2.3^ for infection by strains with a missense mutation in *lrp* or 10^1.8^ for infection by strains with a nonsense mutation.

### Wild-type strains grow faster at the beginning of the infection than *lrp* mutants.

**FIG 3:**
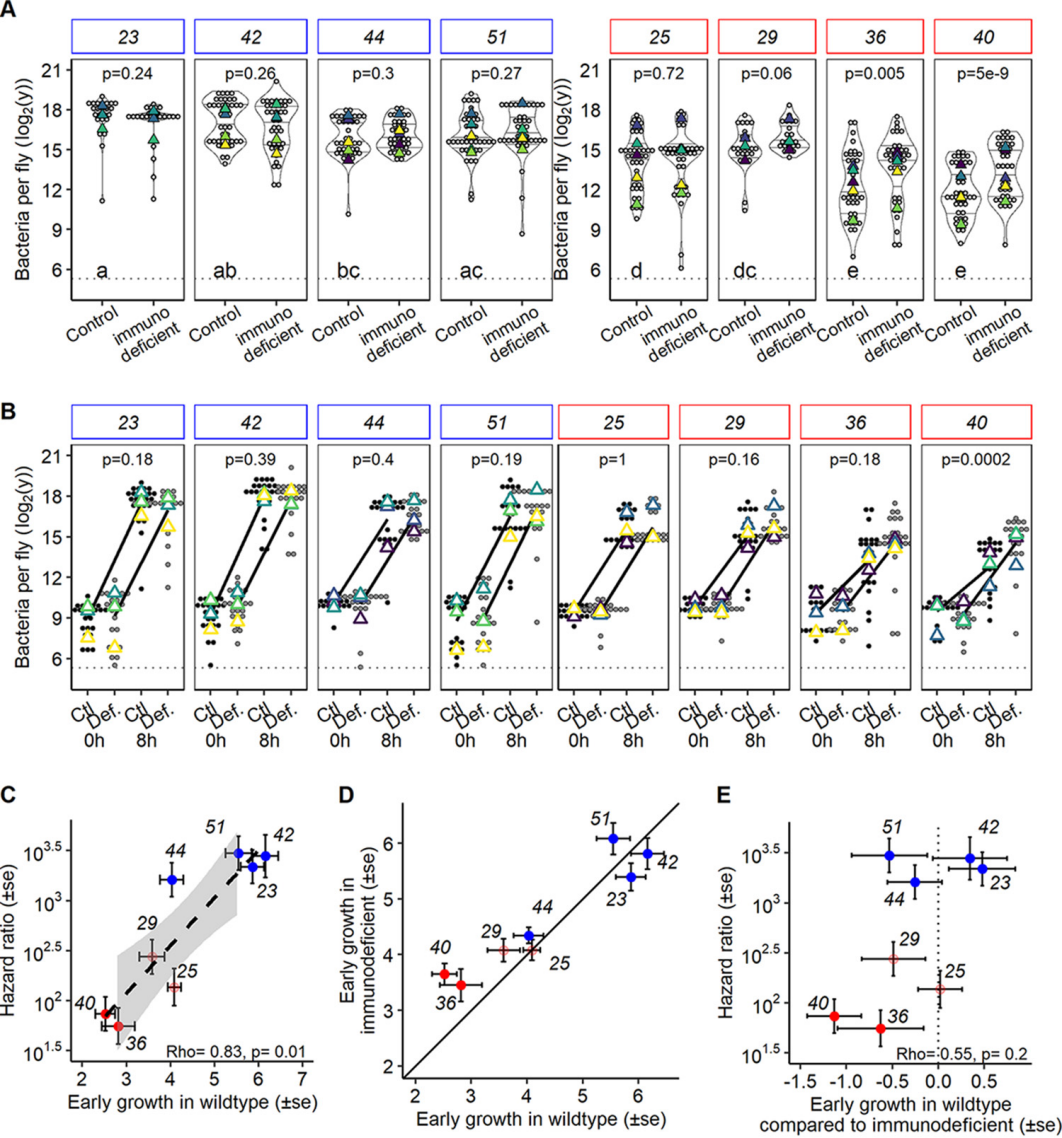
Bacterial success during the first step of the infection. (A) Bacterial load estimated at 8 h postinjection in immunodeficient (IMD) flies (genotype *Dredd^55^*) and in the control genetic background (*w^1118^*). The lowercase letters represent the significant differences between loads of different bacterial strains in hosts with a functional immune system. Wild-type bacterial strains reached a higher density than strains with a mutation in *lrp*. Only mutant strain 29 is intermediate. “p” indicates the *P* value of the difference in bacterial load between immunodeficient hosts and healthy hosts. Unlike other strains, those with a nonsense *lrp* mutation proliferated better in the immunodeficient host. Experiments were replicated with three (strains 23 and 29) or five (all other strains) different overnight bacterial cultures and fly batches. (B) Bacterial load at injection and 8 h postinjection in immunodeficient and healthy hosts. “p” indicates the *P* value of the interaction between time and genotype testing for the difference in proliferation. This approach validates that strain 40, with a nonsense *lrp* mutation, proliferates less in the presence of the immune system. Experiments were replicated with three different overnight bacterial cultures and fly batches. (C) Correlation between early growth in wild-type hosts of the bacterial strains with or without mutation and the hazard ratio (i.e., virulence) extracted for survival analysis in Fig. 2. The virulence correlated strongly with the speed of proliferation at the initiation of the infection. (D) Correlation between early growth in wild-type hosts of the bacterial strains with their early growth in the immunodeficient host. A solid line represents a perfect correlation (i.e., when the immune system does not affect the bacterial growth). Departure from the line (*y* = *x*) indicates a difference in proliferation when the host was immunodeficient. (E) Correlation between the effect of the immune system on proliferation (i.e., estimate of the interaction effect between time and genotype) and the hazard ratio. A dotted line represents the absence of difference between proliferation in immunodeficient hosts and in the healthy host. Values are the estimates extracted from the analysis in panel B. In all panels, blue represents wild-type strains and red represents *lrp* mutants. Colored triangles represent the mean per replicate.

We hypothesized that if wild-type strains are adapted to initiate the infection, they should grow faster than *lrp* mutants in the early stages of the infection. To test this hypothesis, we compared the bacterial loads of hosts infected with four wild-type and four *lrp* mutant *X. nematophila* strains 8 h postinjection. Overall, hosts infected with wild-type strains had higher bacterial loads 8 h postinjection (fitme mutation effect, df = 1, likelihood ratio test [LRT] = 153.39, *P* = 3.13e−35 [Fig. 3A]). After only 8 h of infection, strains with a nonsense mutation in *lrp* were already four divisions or more (i.e., 4 units in log_2_) behind other strains (median log_2_ bacterial load: nonsense mutants, strain 36 = 11.5 and strain 40 = 11.6; other strains, strain 23 = 18, strain 42 = 17.2, strain 44 = 15.5, strain 51 = 15.7, strain 25 = 14.6, and strain 29 = 15.1 [Fig. 3A]). Only mutant strain 29, bearing a missense mutation, had a nonsignificantly lower load than wild-type strains after 8 h of infection. We tested if the difference in bacterial loads between wild-type and mutant strains was linked, at least in part, to the host immune response. As a Gram-negative bacterium, the main immune pathway activated by *X. nematophila* infection in *Drosophila* is the IMD pathway. The IMD pathway is so important in controlling Gram-negative infections that individuals lacking the pathway can die in few hours from infections that are otherwise benign (42, 43). It is already known that *X. nematophila* triggers a sustainable IMD response upon systemic infection, that immunodeficient hosts die earlier, and that hosts immune primed by other Gram-negative bacteria survive longer during subsequent infection by *X. nematophila* (39, 40). Thus, we compared the bacterial load in control healthy hosts to that in hosts carrying a knockout mutation in *Dredd*, a critical gene for the activation of the IMD pathway (44), at 8 h postinjection. We found that bacterial strains with a nonsense *lrp* mutation had a significantly higher bacterial load in immunodeficient hosts than in healthy hosts, while wild-type and missense bacterial strains had the same bacterial load (Fig. 3A). To further support the result suggesting that nonsense mutations in the *lrp* gene trigger a susceptibility to the host immune system, to which wild-type strains seem to be fairly insensitive, we compared the difference in growth rates between 0 and 8 h postinjection (Fig. 3B). The impact of IMD expression on bacterial proliferation can be quantified as an interaction term in a statistical analysis of bacterial load that includes host genotype (healthy versus immunodeficient) and time (0 versus 8 h) as explanatory factors. We found that the interaction was strongly significant only for one of the two nonsense mutations (strain 40 [Fig. 3B]). However, even if not significant when tested overall, we found lower proliferation for the other strain with a nonsense mutation (strain 36) in two out of three replicates (Fig. 3B). The virulence, expressed as a higher hazard ratio, correlated with the ability to grow at the beginning of the infection (Fig. 3C). However, even if nonsense mutations proliferated better in immunodeficient than in healthy hosts (Fig. 3D), the ability to proliferate with the immune system, as expressed as the difference in proliferation in healthy and immunodeficient hosts (i.e., the statistical interaction mentioned above), did not correlate with the virulence (Fig. 3E).

### *lrp* mutations do not alter bacterial pathogenicity.

**FIG 4:**
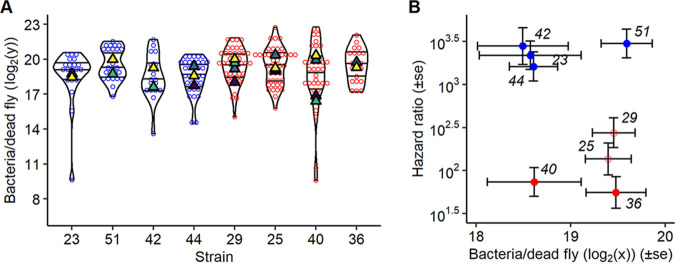
Bacterial load upon death (BLUD) estimated approximately 12 h to 30 h after injection in wild-type hosts. (A) Mutant strains reached the same BLUD as wild-type strains. Experiments were replicated with three different overnight bacterial cultures and fly batches. (B) Correlation between BLUD and hazard ratio. Mutant and wild-type strains reached the same BLUD, but wild-type strains had a higher hazard ratio and reached the BLUD about 10 h earlier, suggesting that *lrp* mutations affect virulence but not pathogenicity. Colored triangles represent the mean of the replicates.

The mutations could change virulence via a change in bacterial pathogenicity (i.e., the ability to kill at a given load). We investigated the role of the mutation on pathogenicity by estimating the maximal bacterial load a host can sustain before dying (i.e., the bacterial load upon death [BLUD]; see reference 2). For a given host genotype with a fixed tolerance, the variation in BLUD when infected with different bacterial strains reflects the different amounts of damage these strains cause to their host. In that case, the BLUD can thus be considered a proxy measure of pathogenicity. We hypothesized that the *X. nematophila* wild-type strains, which kill faster, would be more pathogenic (e.g., secrete more toxins) than the mutants, and thus, the hosts would succumb to a lower load with wild-type strains than with mutant strains: i.e., the BLUD would be lower in hosts infected by wild-type strains. We found that even if hosts died earlier from infections with wild-type strains than with *lrp* mutant strains, the BLUD was the same (fitme, df = 1, LRT = 2.51, *P* = 0.11 [Fig. 4A]). As even nonsense mutations had the same BLUD as the wild type, it is likely that *lrp* does not have a role in the pathogenicity of *Xenorhabdus*. This is further suggested both by the absence of correlation between the mean of the hazard ratio of a strain, a proxy for its virulence, and its BLUD mean (Fig. 4B) and by previous results showing that *lrp* mutation does not abolish *in vitro* insecticidal secretion activity (39).

### *lrp* mutants are better at proliferating in the dead host.

**FIG 5:**
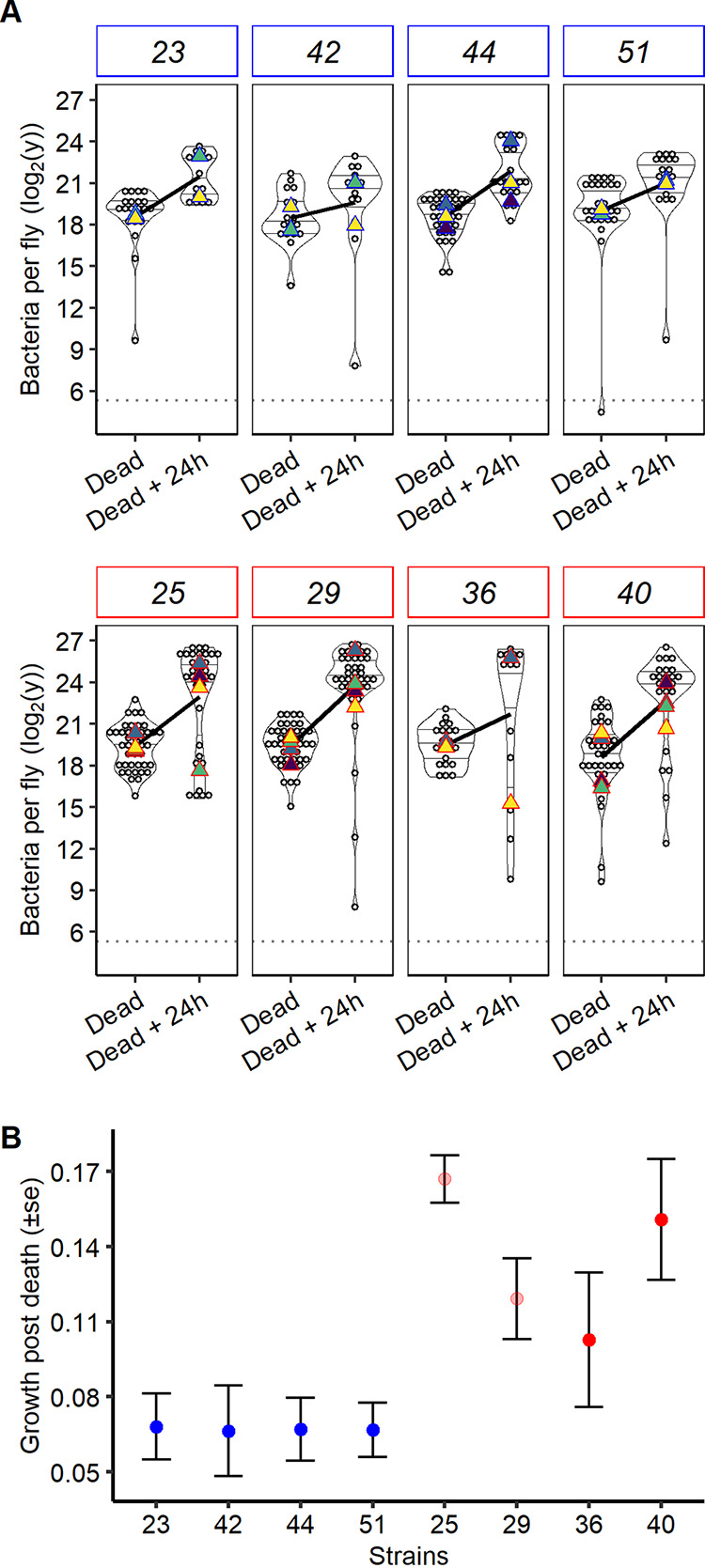
Proliferation during the second step of infection, within the dead host. (A) Proliferation between death and 24 h later in the wild-type host and (B) model estimate of growth postdeath (i.e., estimate of the parameter “time” in the model to analyze panel A). Unlike when the host was alive, *lrp* mutant strains had a higher density than wild-type strains when the host was dead. Missense mutation strains (light red) did not proliferate differently in dead hosts from nonsense mutation strains (dark red). Experiments were replicated with two (strains 23, 36, 42, and 51), three (strain 44), or four (strains 25, 29, and 40) different overnight bacterial cultures and fly batches.

We investigated the difference in growth rates in dead hosts by estimating first the BLUD and then the load 24 h later. Assuming that *lrp* mutants are selected by the new environment associated with the host’s death, we hypothesized that they would proliferate better during these 24 h. All strains were able to grow in dead hosts; however, mutants had a much better ability to grow under those conditions than did wild-type strains (Fig. 5A). Interestingly, carrying a missense mutation in *lrp* was sufficient for a strain to be better adapted to this condition, growing at a comparable rate to strains carrying nonsense mutations (Fig. 5B).

### Trade-off step-specific adaptations due to *lrp* mutations.

**FIG 6:**
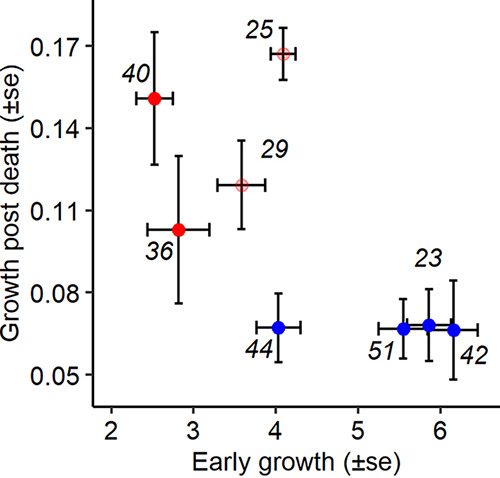
Trade-off between proliferation in the first and second steps of infection. The correlation between proliferation at the start of the infection and within the dead host is shown. While wild-type strains proliferated best at the initiation of the infection, they performed poorly in the dead host. Conversely, *lrp* mutant strains proliferated better in the dead host but lost the ability to proliferate rapidly at the initiation of the infection.

There was a clear qualitative trade-off between the ability to proliferate early in the infection and the ability to proliferate in the dead host (Fig. 5B and 6). The type of mutation did not determine this trade-off, and even if there was a strong trend, we could not quantitatively show a negative correlation between those two abilities (rho = −0.67, df= 6, *t* = −2.2 h, *P* = 0.06 [Fig. 6]). This suggests that changes to, or abrogation of, Lrp function improve performance during the waiting step of the infection but drastically reduce performance at the initiation of the infection.

## DISCUSSION

At each step of an infection, infected individuals can potentially stop the progress of their parasites. Consequently, there is strong selection on parasites to reach the next step and complete their life cycle (3, 45). However, the traits allowing the parasite to progress through each step may be strikingly different and sometimes even be mutually costly. Using D. melanogaster as the host and Xenorhabdus nematophila as a bacterial pathogen, we showed that indeed the bacterial physiological requirements for successive steps of infection are different and can trade off.

In the system, Xenorhabdus nematophila (bacterium)-Steneirnema carpocapseae (nematode)-insect, the nematode vector injects the bacteria into the insect host body cavity to kill it and then uses the nutrients of the dead host to reproduce. At the beginning of the infection, bacteria proliferate in a given environment, where they need to be highly virulent to prepare the conditions for their vector; once the host is dead, the environment necessarily changes (e.g., in terms of nutrients, oxygen concentration, or pH level), and bacteria need to optimize their use of resources that are not replenished until the nematodes produce dispersing larvae. Part of this optimization is accomplished by gene regulation. But over the course of an infection, mutations also occur in *X. nematophila* populations. The mutations in *lrp* increase in frequency in the population (7), but if they potentially can be carried by the vector, they reassociate poorly (16, 39)—possibly because *lrp* affects the expression of genes involved in the mutualism (46). This suggests that wild-type strains, which are those found in wild-caught founder nematodes (18, 47), are adapted to disperse and initiate the infection, while mutants are adapted to persist in the host. We found that both wild-type strains and *lrp* mutants proliferated faster *in vivo* at the step to which they were expected to be better adapted.

The control of bacterial proliferation by the Drosophila melanogaster immune system can take several hours (2), and the acquired immune system has evolved to reduce this crucial time where bacteria proliferate exponentially if untended. Hence, to increase their chance for a successful infection, bacteria can adapt to either suppress, resist, or outpace the humoral immune response (i.e., the control via antimicrobial peptide [AMP] secretion) by killing the host before the AMP concentration is high enough to control its proliferation. Our results show that wild-type strains are most likely better adapted to start the infection because (i) they proliferate faster in the first 8 h of the infection, (ii) they do so at the same speed with or without the host immune response, unlike most *lrp* mutants, and (iii) they kill hosts at the same load as *lrp* mutant strains, but do so 10 h earlier. The way in which wild-type bacteria are better adapted to the first step of the infection is still not entirely clear. The efficacy of *Drosophila* cellular immunity in the adult is likely to depend on the infection and is regularly weak (2, 48). Therefore, *Drosophila* might not have a sufficiently strong cellular immune system to control a virulent pathogen such as *X. nematophila*, unlike the cellular immunity of a lepidopteran. For this reason, our study may underestimate the potential adaptation of *X. nematophila* to cellular immunity. Yet, when the *Drosophila* humoral immune response is activated by preexposure to an avirulent bacterium, *X. nematophila* is sensitive to the circulating AMPs (39, 40). This suggests that AMPs can control the infection and that *X. nematophila* is not specifically resistant. However, *X. nematophila* is known not only to immunosuppress cellular immunity in lepidopteran larvae (49, 50) but also to downregulate cecropin, an AMP (51). Hence, it is likely that wild-type bacteria delay the humoral immune response to kill the host faster. However, *lrp* mutants still kill their host relatively quickly, and the ability to grow despite the immune response did not correlate quantitatively with the death hazard ratio, which suggests that the efficiency to immunosuppress the host is not the main reason for the higher virulence of the wild type. However, the death hazard ratio correlated with the speed of proliferation during the first step of infection, which suggest that the adaptation to this step is mainly the intrinsic proliferation rate in this environment.

Although the mutants proliferate in larger numbers in dead hosts, showing that they are better adapted to the second step of infection, they reassociate poorly with the nematode (39), and in addition, this adaptation traded off with the ability to grow at the first step of the infection. If they are not found in a wild-caught nematode and are not good at initiating an infection, it is not trivial to understand how mutants are so prevalent in the system. The *lrp* mutants could be selected inside the body of their host, during the step of proliferation, but would not have an advantage over the whole transmission cycle, as they do not favor a good transmission. This phenomenon is reminiscent of the short-sighted selection of cancerous cells within their host. However, because wild-type strains with mutant offspring would have lower reproductive success, it is likely that any mechanisms preventing those mutations would be advantageous. Alternatively, the occurrence of the mutants may still be important in the symbiosis if they give an advantage to their kin wild-type strains. Indeed, *lrp* mutants are likely to modify the nutritional value provided by a dead host, and because they can grow in higher numbers in the dead host, they can feed the vector by being preys. Hence, by feeding the vectors directly or indirectly, the mutants may sustain the system and allow the nematode to disperse with a kin wild-type strain or with a mutant that would have reverted its phenotype.

In our system, the step transition between persistence in a living host and in a dead host is an illustration of the different transitions that can happen in many other diseases and how trade-offs between step-specific adaptations can occur. The change occurring between the acute phase of an infection and its chronic phase is similarly one important and common transition over the course of an infection. One of the bacterial adaptations to this transition is known as the small colony variant (SCV). SCVs are important for the chronic establishment of many human diseases, such as, for instance, those associated with Staphylococcus aureus, Pseudomonas aeruginosa, *Salmonella* serovars, Vibrio cholerae, Brucella melitensis, *Shigella* spp., or Neisseria gonorrhoeae (reviewed by Proctor et al. [52]). The pathogen Staphylococcus aureus is a good example to illustrate the selection intrahost of SCVs as it is its ability to change rapidly from an extracellular aggressive state to an SCV adapted for intracellular infection that allows for long-term persistence in its host. The typical S. aureus SCVs form small colonies on agar plates, have decreased respiration, pigmentation, hemolytic activity, and coagulase activity, increased resistance to aminoglycoside, and an unstable colony phenotype (52). *In vivo*, SCVs usually gain in fitness by acquiring the ability to establish and remain chronic in the host after they have gone through the acute phase of the infection. The phenotype of *X. nematophila* that is selected during the waiting step (i.e., that of the mutant strains of our study) is very similar to the commonly described SCV (7, 19). In fact, an SCV was observed in *X. nematophila* but not identified as such (53), and the term has been used to describe the alternative phenotype of a closely related species, Photorhabdus luminescens, which also needs to wait in the dead insect until its nematode vectors disperse (54–56). The biochemical basis of SCVs in mammalian pathogens is generally a single or multiple auxotrophism caused by a simple genetic alteration, exemplified by mutations of genes involved in the biosynthesis of thiamine, menadione, hemin, or thymidine (57). Mutations in *lrp* have indeed been found to be involved with induced auxotrophy in E. coli and Komagataeibacter europaeus (58, 59) and with the SCV phenotype in Pseudomonas aeruginosa (60). Likewise, mutation in *lrp* is sufficient to switch to SCV-like phenotypes in *X. nematophila*, allowing it to persist in the dead host until the vector disperses. Hence, we believe that our bacterium-insect model system allowed us to study the advantage that SCV can provide over the course of an infection and the trade-off between adaptations of infection steps. As such, it suggests that the bacteria initiating a disease can be drastically different from the bacteria selected within the host at later stages of infection and that intrahost selection is a factor to take into account to understand a pathogen and its weakness during an infection.

## MATERIALS AND METHODS

### Fly stocks and husbandry.

Drosophila melanogaster flies were reared on flour-yeast medium (containing the following per liter of water: 70 g of corn flour, 70 g of yeast, 15 g of agar, 10 g of vitamins [Vanderzant vitamin mixture for insects from Sigma-Aldrich], 10 g of tegosept diluted in 20 ml of ethanol, and 3 g of propionic acid). Males between 3 and 9 days old (age was standardized within each experiment) were used in all experiments. Rearing and experiments were conducted at 25 ± 1°C with a 12-h/12-h light/dark cycle. We used Canton S flies and *w^1118^* inbred, laboratory strains as wild-type genotypes. To test for the susceptibility of the bacteria to the immune system, we used an immunodeficient host line in which IMD pathway signaling is impaired due to a mutation in the gene *Dredd^D55^* (61). The levels of antimicrobial peptides in response to infection of *Dredd^D55^* flies, backcrossed in the *w^1118^* genotype, have not been quantified in our study but have been shown to be much lower in several previously ones (e.g., see reference 62). This *Dredd* allele has even been confirmed recently by resequencing. It is at X:634,862. A G-to-A nucleotide substitution alters the codon from TGG (tryptophan [W]) to TAG (Stop), which produces a truncated protein (personal communication by François Leulier to FlyBase, no. FBrf0243539).

### The bacterium.

Xenorhabdus nematophila is generally described as having two distinct phenotypes, distinguished by their capacity to adsorb bromothymol blue. When plated on a bromothymol agar (NBTA) nutrient (15 g of nutrient agar, 3 g of beef extract, 5 g of peptone, 8 g of NaCl, 0.04 g of triphenyl 2,3,5-tetrazolium chloride [TTC], and 25 mg of bromothymol blue per liter of water), wild-type bacteria form blue colonies, while others form red colonies. This phenotype is associated with mutations in the *lrp* gene, which the wild-type strains do not carry (7, 19, 34).

Our strains were chosen from a collection of 34 strains isolated from independent *in vitro* culture after several days of a prolongated nonagitated growth in LB medium (Luria broth, Miller). (See the description of the strains in reference 7.) Although this well-characterized collection was obtained from *in vitro* culture, similar mutants have been found *in vivo* (7). Strains 21, 23, 42, 44, and 51 are wild type for the gene *lrp*. *lrp* is composed of two domains: a DNA-binding domain called HTH and a ligand-binding domain called RAM (63) The latter generally interacts directly with amino acids, while the former, in response to amino acid concentration, interacts with DNA to modify the expression of hundreds of genes (21, 22). Strains 25 and 29 have a missense mutation (i.e., a single base pair change in the domain RAM of *lrp*, both at codon position 120 in single nucleotide polymorphisms [SNPs] 358 and 359, respectively, leading to amino acid substitutions). Strains 36, 39, and 40 have an indel mutation in *lrp*. The first two have a frameshift in the HTH domain (at codon position 53), while 40 has a nonsense mutation (i.e., a 26-bp insertion in SNP 372 leading to a Stop codon) in the RAM domain. Hence, while mutations in strains 25 and 29 are expected to affect the function of the protein, mutations in strains 36, 39, and 40 are expected to stop its function completely. (Details are also summarized in Fig. 1.) To summarize, we used two strains of *X. nematophila* per mutation type, such that our results will not be particular to one genotype.

### Quantification of bacterial suspension.

*X. nematophila* was grown in liquid LB medium. Overnight cultures, started from a single bacterial colony, were grown to saturation at 28°C under agitated conditions (180 rpm). To prepare the suspension used for injection, we first centrifuged the culture (10,000 rpm for 5 min), discarded the supernatant, and resuspended the bacterial pellet in 1 ml of LB, measured the optical density at 600 nm (OD_600_) by spectrophotometer, and adjusted by dilution to an OD_600_ of 0.1. To standardize the quantities of bacteria injected inside the host, and because the wild-type and mutant strains differ in their absorbance, we also used a Thoma cell counting chamber to enumerate cells under a microscope (Olympus BX 51, magnification of ×200).

### Xenorhabdus nematophila injection in Drosophila melanogaster.

We injected approximately 1,000 bacteria per fly in 23 nl of LB medium, between the two first abdominal segments, using a Nanoject 2 injection system (Drummond) (64). Controls were injected with 23 nl of sterile LB medium. Prior to injection, flies were anesthetized with CO_2_. Anesthesia lasted for about 5 min, and flies were observed afterward to confirm they recovered from the procedure.

### Host survival.

Upon injection of approximately 1,000 bacteria, ∼50 flies per replicate were kept in 900-ml plastic boxes at 25°C with 60% humidity and with *ad libitum* access to food. Survival was scored hourly, starting around 10 h postinjection. The dose of bacteria injected was chosen with the rationale that it was within a realistic range for an initial load with respect to natural infections, but high enough to be sure that each host was exposed to the bacteria, as very low doses are more prone to random variation in bacterial number, with in some cases no bacteria being injected. All flies died rapidly upon injection, showing that they were all exposed.

### Estimation of bacterial load.

To characterize the bacterial within-host dynamics, we quantified the bacterial load in individual flies at two time points during the infection. To extract bacteria from the host, we homogenized individual flies at 30 Hz for 1 min using a tissue lyser (Tissue Lyser II) in 250 µl of LB medium containing two 2-mm-diameter glass beads. At least eight independent replicate measurements (i.e., separate extractions on 8 individual flies) were performed per time point, treatment, and experiment. Samples were serially diluted 1 to 1:2,500 in 96-well microplates. We then deposited 5-µl drops from each well onto an NBTA plate using an Integra Viaflo 96 micropipette. Plates were incubated for 48 h at 28°C, and then we counted the number of colonies that grew within each drop. Such raw data were used in the analysis, but for graphical display, we instead used estimations of bacterial loads per fly obtained by adjusting a Poisson generalized linear model, where the number of CFU is predicted from dilution. To estimate the bacterial load upon death (BLUD) (2), infected hosts were checked every 30 min, and newly dead flies were collected and homogenized, with bacterial load quantified as described above (2). Bacterial load 24 h after the host death was quantified from individuals kept in closed sterile microtubes. To estimate early growth in the wild type and growth postdeath, we extracted the estimates of the effect of the variable time and their standard error from the models used to analyze the growth of each strain. To estimate early growth in the wild-type compared to immunodeficient strain, we extracted the estimates of the interactions between time and D. melanogaster lines from the model used to analyze the difference in growth in the control line versus immunodeficient line of each strain.

### Statistical analyses.

We carried out all analyses using R (65). We analyzed differences in survival (time-to-death curves) using the R packages Survival and coxme (66). We used the coxme routine, which allows inclusion of random effects in a Cox model, to model variability among day of experiments and replicated vials. We determined how host survival is affected by bacterial mutations by comparing a Cox model including mutation status as a fixed effect to a model without this fixed effect. We extracted the hazard ratio from this survival model to compare survival among strains, taking into account experimental replications. We analyzed the differences in bacterial load within the host using general linear mixed models (GLMM) implemented in the package spaMM with the function fitme (67). We tested the effect of mutation on bacterial load within the host by comparing raw data (i.e., the counts of bacterial colony [CFU] in a 5-µl drop over several dilutions—only in drops containing less than 50 CFU). Dilution and volume were included in the model as fixed offsets. As before, we modeled fluctuations among experiments or replicate variants as random effects. An additional random effect was added to take into account the count uncertainty among drops for the same host.
